# Randomized, placebo-controlled trial reveals the impact of dose and timing of *Bifidobacterium infantis* probiotic supplementation on breastfed infants’ gut microbiome

**DOI:** 10.1128/msphere.00518-25

**Published:** 2025-12-22

**Authors:** Claire E. O'Brien, Steven A. Frese, Karina Cernioglo, Karla Damian-Medina, Ryan D. Mitchell, Giorgio Casaburi, Ryan A. Melnyk, Bethany M. Henrick, Jennifer T. Smilowitz

**Affiliations:** 1 Health and Environmental Sciences Institute 460456, Washington, DC, USA; 2 Department of Nutrition, University of Nevada 206786, Reno, Nevada, USA; 3 Reno School of Medicine, University of Nevada 12290, Reno, Nevada, USA; 4 Department of Nutrition, University of California Davis 8789, Davis, California, USA; 5 PumpKin Baby Inc., Princeton, New Jersey, USA; 6 InnerPlant Inc., Davis, California, USA; 7 Exagen, Inc., Vista, California, USA; 8 Lawrence Berkeley National Laboratory 1666, Berkeley, California, USA; 9 Department of Food Science and Technology, University of Nebraska 14719, Lincoln, Nebraska, USA; University of Michigan Medical School, Ann Arbor, Michigan, USA

**Keywords:** *Bifidobacterium infantis*, breastfed, infant gut microbiome, probiotic, randomized, placebo-controlled trial

## Abstract

A dysfunctional gut microbiome has become increasingly common in infants born in high-income countries as *Bifidobacterium* strains no longer dominate the gut microbiome. Probiotics containing *Bifidobacterium infantis* have been used in breastfed newborns to successfully restore the gut microbiome; however, no studies to date have demonstrated this effect in older breastfed infants whose gut microbiomes are transitioning toward stability and maturity. This is a 9-week randomized controlled trial wherein 2–4 months old exclusively breastfed infants (*n* = 40) received 0 CFU/day *B. infantis* EVC001 (placebo), 4.0 × 10^9^ CFU/day *B. infantis* EVC001 (low), 8.0 × 10^9^ CFU/day *B. infantis* EVC001 (medium), or 1.8 × 10^10^ CFU/day *B. infantis* EVC001 (high) in equal allocation for 28 consecutive days beginning on day 8. Stool samples were collected on study days 7, 10, 14, 21, 28, 35, 42, and 63. Fecal *B. infantis* levels were significantly higher in all supplement groups compared with placebo on day 28 and day 63. On day 28, fecal *B. infantis* levels were significantly higher in infants who received any (low, medium, and high) dose compared with baseline. The abundance of fecal *Bifidobacteriaceae* significantly increased nearly 2-fold in response to *B. infantis* EVC001 supplementation. No matter the dose, probiotic supplementation with *B. infantis* in 2- to 4-month-old exclusively breastfed infants resulted in colonization until at least 1 month post-supplementation.

**IMPORTANCE:**

This study found that supplementing exclusively breastfed infants with a probiotic, Bifidobacterium infantis EVC001, between 2 and 4 months of age can successfully restore beneficial bacteria in their gut, even after the newborn period. Although previous research showed this effect in newborns, this is the first study to demonstrate that older infants, whose gut microbiomes are typically more stable, can still benefit. The probiotic was effective at all tested doses, with higher levels of *B. infantis* and overall Bifidobacteriaceae in infants' stool during and even 1 month after supplementation. This study demonstrates that *B. infantis* can take hold in the gut and potentially improve gut health in older breastfed babies, offering a promising approach to support infant health in settings where beneficial gut bacteria are often missing.

**CLINICAL TRIALS:**

This study was registered at clinicaltrials.gov as NCT03476447.

## INTRODUCTION

Human milk plays a critical role in the development of a healthy infant gut microbiome in the weeks and months after birth, providing both nutrition and protection to support the development of the vulnerable neonate. It delivers a wide spectrum of biologically active molecules that aid in the development and maturation of the gut, the innate and adaptive immune systems, and support the growth of a protective gut microbiota, namely, *B. infantis*, a subspecies of *Bifidobacterium longum* (1, 2). Specifically, human milk oligosaccharides (HMOs), an abundant component of human milk that is not digestible by humans, act as prebiotics that selectively enrich the growth of *B. infantis*. Unlike other bacteria found in the infant gut, *B. infantis* is uniquely adapted to the breastfed infant gut. This subspecies possesses five distinct gene clusters that encode a set of glycoside hydrolases and oligosaccharide transporters, which enable it to efficiently metabolize HMOs with a degree of polymerization of 7 or less (3) and in a strain-specific manner (3–7). The byproducts of HMO metabolism, lactate and acetate, decrease intestinal pH, which has been shown to inhibit the growth of potential disease-associated bacteria and increase intestinal barrier function (2, 8).

Data from non-industrialized populations suggest the historical norm of the breastfed infant gut microbiome is dominated by *Bifidobacterium,* particularly *B. infantis*, during the first 6 months of exclusive breastfeeding (9, 10). However, in recent decades, the dominance of fecal *Bifidobacterium* has declined in high-income countries, as evidenced by an increase in fecal pH from 5.0 to 6.5 over the past 100 years (11). Multiple factors, including geography (9, 10), industrialization (9, 10), delivery mode (12, 13), and infant feeding practices (10), have profound impacts on the developing gut microbiome. The unintended consequences of certain medical practices, such as cesarean section delivery (12, 13), infant formula feeding (13), and antibiotic use, disrupt normal gut microbial development (14). A recent cross-sectional analysis by Taft and colleagues demonstrated that horizontal transfer plays a critical role in the transmission and colonization of *B. infantis*, which was positively associated with breastfeeding duration (10). The loss of *B. infantis* is implicated in a dysfunctional gut microbiome and associated with increased risk to atopic diseases (15). Studies in Bangladeshi breastfed infants have reported correlations between higher levels of fecal *B. infantis* and stronger antibody responses to vaccines (16, 17). Thus, re-establishing *B. infantis* in the gut microbiome early in life may be key for supporting gut and immune health.

A dysfunctional gut microbiome in infants can be mitigated with supplementation of a probiotic containing *B. infantis* combined with a human milk diet. Administration of *B. infantis* in preterm infants resulted in increased relative abundance of fecal bifidobacteria, with the highest fecal levels in breastfed infants (18). The results from a clinical trial have demonstrated that supplementation with a high dose of *B. infantis* (1.8 × 10^10^ CFU/day) in breastfed newborns leads to the stable colonization of *B. infantis* in the infants’ gut at 1 month (19) and 1-year post-supplementation, even during complementary feeding as long as human milk was also part of the infants’ diets (20). Additionally, supplementation significantly increased fecal short-chain fatty acids and decreased the fecal pH and residual fecal HMO concentrations, which suggests a higher consumption of HMOs by *B. infantis* and changes in intestinal fermentation (19). A dominance of fecal *Bifidobacterium* in response to *B. infantis* supplementation also reduced the abundance of antibiotic-resistant bacteria (21) and significantly reduced enteric inflammation (22). Henrick et al. showed that fecal waters from infants colonized with *B. infantis* polarized naïve CD4+ T cells toward intestinal T helper 1 (T_H_1) phenotype, whereas fecal waters from infants not colonized with *B. infantis* skewed T cells toward T_H_2 and T_H_17 phenotypes, demonstrating a functional link between beneficial gut microbes and immunoregulation during the first months of life (23). The impacts of immunoregulation in early life may have practical implications on immunity throughout childhood (15–17). Thus, *B. infantis* probiotic supplementation combined with a human milk diet may be a viable tool to combat infants’ dysfunctional gut microbiome, thereby facilitating proper immune system and intestinal development.

To date, no studies have determined the effectiveness of a probiotic supplement in colonizing the intestinal tract in older infants or compared its impact across different doses. As intestinal microbial communities become more stable and complex over time, it is unclear if probiotic supplementation with *B. infantis* in older infants results in similar effects on microbial colonization compared with newborn infants (13, 24). Therefore, it is important to identify whether *B. infantis* administration can also successfully establish colonization of healthy microbial species and ensure the persistence of this microorganism in older infants.

To address this question, we conducted a randomized, double-blind, placebo-controlled study to (i) determine if *B. infantis* supplementation in older (2–4 months old) exclusively breastfed infants results in the stable colonization of *B. infantis* in the gut and (ii) determine the minimally effective dose of *B. infantis* supplementation needed to increase the abundance of *B. infantis* to levels similarly found in exclusively breastfed newborns. We hypothesized that infant fecal *B. infantis* during supplementation would be higher with *B. infantis* EVC001 supplementation compared with placebo at day 28.

## MATERIALS AND METHODS

### Study population

Between April 2018 and March 2019, healthy women who had recently delivered healthy full-term infants and lived within the Davis and Sacramento metropolitan region of northern California (USA) were recruited to enroll in this study. Inclusion criteria for study participants were as follows: healthy women 21 years of age or older; healthy infants born full-term (greater than 37 weeks gestation) without medical complications who were 60–125 days old at time of enrollment; infants exclusively breastfed with maternal intent to continue exclusive breastfeeding for at least 9 additional weeks following study enrollment; mothers who were willing to refrain from feeding their infants infant formula, solid foods, and iron or non-study supplements before the end of the study period. Exclusion criteria for study participants were as follows: infants born in a multiple birth; infants who had taken antibiotics, iron supplements, or consumed infant formula or *Bifidobacterium*-containing probiotics within 4 weeks of enrollment or during the baseline period; infants who consumed any probiotics containing *B. infantis* since birth; infants who consumed any solid food since birth; mothers who consumed probiotics containing *B. infantis* during the 3rd trimester of pregnancy and gave birth vaginally; and mothers who smoked cigarettes during pregnancy, were currently smoking, or who planned to resume smoking during the study period.

### Study design

The study duration was 9 weeks and consisted of a 1-week baseline period (days 1–7), a 28-day supplementation period (days 8–35), and a 28-day post-supplementation period (days 36-65). After meeting the study criteria, participants provided written informed consent. On study day 7, participants underwent final screening by interview for the consumption of infant formula, antibiotics, probiotics, iron supplements, solid foods, or beverages other than human milk or water and were randomized into one of four supplement groups.

Randomization was generated through Statistics & Data Corporation (SDC) (Tempe, Arizona) utilizing Interactive Response Technology built into a clinical data management system (iMedNet), and study personnel were blinded to the treatment allocation. Because delivery mode has been shown to confound the infant gut microbiome, participants were stratified to one of two randomization schemes based on mode of delivery—vaginal or cesarean section (13, 14, 25). Randomization to supplementation of 0 CFU/day *B. infantis* EVC001 (lactose placebo), 4.0 × 10^9^ CFU/day *B. infantis* EVC001 (low dose), 8.0 × 10^9^ CFU/day *B. infantis* EVC001 (medium dose), or 1.8 × 10^10^ CFU/day *B. infantis* EVC001 (high dose) was in equal allocation (1:1:1:1 ratio) for all randomization schemes. The medium dose was selected because this dose is commercially available for probiotics sold in the United States. The high dose was selected because it delivers ~2× more colony-forming units than the medium dose and has been shown to effectively restore the gut microbiome of human milk-fed newborns (19). The low dose was selected because it delivers half the number of colony-forming units compared with the commercially available, medium dose. The placebo and *B. infantis* EVC001 supplements were provided by Infinant Health Inc. (Davis, CA). Each *B. infantis* EVC001 supplement was made up of a blend of the probiotic and lactose. Each supplement sachet contained 625 mg of blended powder.

A total of 41 participants were enrolled in this study; however, only 10 infants were randomized into each group. One participant withdrew from the study prior to randomization, and one participant from the 8.0 × 10^9^ CFU/day (medium dose) *B. infantis* group withdrew from the study during the Supplementation period; thus, only nine subjects received the 8.0 × 10^9^ CFU/day (medium) *B. infantis* supplement.

This study is powered based on the minimum effective dose to increase fecal *B. infantis* compared with a placebo control in exclusively breastfed infants. Based on day 21–30 infant fecal *B. infantis* levels from the IMPRINT study (19), a minimum sample size of four infants in each dosing group was needed to identify a 9.7-log difference, with an α = 0.01 (to account for multiple testing within each family of hypotheses), and power = 90%.

Infants received one daily serving of the study supplement (lactose placebo, low, medium, or high doses) for 28 consecutive days beginning on day 8 and continuing through day 35. During the day 7 randomization visit, mothers were trained by study personnel to mix the contents of each supplement sachet with approximately 5 mL of their breast milk in a plastic medicine cup, and to syringe-feed the mixture to their infants at home. The product was stored in a freezer at −20°C at the UC Davis campus until distributed to study participants. Mothers received 28 sachets, plus four extra sachets to be used in the event of damage or misplacement. All sachets were kept frozen in the mothers’ kitchen freezers until the time of use. Participants were instructed to keep all used and unused sachets provided. Compliance was assessed on day 37 by recording the number of used and unused *B. infantis* sachets. Compliance was defined as at least 21 doses (75% of the scheduled supplementation) of the randomized study supplement (19). Study compliance was calculated as the percentage of supplement sachets used out of the 28 provided, using the formula: (number of used sachets ÷ 28) × 100.

Participants were followed from day 36 to day 65 to assess the persistence of *B. infantis* 1 month post-supplementation. Following completion of the study on day 65, all participants were offered a 28-day supply of Infinant Health’s commercially available *B. infantis* probiotic product (Evivo). Infant weight was measured by study personnel with a digital infant scale (Tanita) on days 7, 37, and 65.

### Questionnaires

At the enrollment visit on day 0, mothers completed questionnaires regarding their pregnancy, labor, and delivery experience, reproductive health, and their infant’s health and diet since birth. At the subsequent three study visits, mothers completed questionnaires about their and their infant’s health and diet since the previous study visit. On each day throughout the entire study, participants were asked to keep prospective, daily logs regarding their infant’s general health, stool patterns (stool number, size, and consistency (26), diet, and medication usage. Participants were asked to record all periods of sleep, crying, and fussing (minutes) for two 24 h periods (the two 24 h periods did not have to be consecutive) during baseline (days 1–6) and the supplementation period (days 21–28) if they lasted for 5 min or longer.

### Samples

Fecal samples were collected at home by parents from their infant’s diapers before study day 7 (baseline), and on study days 10, 14, 21, 28, 35, 42, and 63 using PurFlock Ultra Flocked Swabs (Puritan, Guilford, ME) and DNA/RNA Shield Lysis and Collection Tubes (Zymo Research, Irvine, CA). Additional fecal samples were collected before study day 7 and on days 14, 21, 28, and 63 using a Burkle SteriPlast micro spatula (VWR, cat # 75876-080) and a disposable cosmetic spatula (Pana Brand). Feces were placed into a 5 mL Eppendorf tube (VWR, cat # 89429-310) and initially stored in participants’ home freezers. Fecal samples were transferred by participants to the University of California Davis in insulated cooler bags with ice packs during the study visit days: 7, 37, and 65. Upon arrival at the University of California Davis, the samples were visually inspected for thawing by study personnel and immediately stored in a −80°C freezer. All individuals who processed and analyzed the samples were blinded to treatment allocation.

### Molecular methods and analysis

As previously described (19, 20), total DNA was extracted from ~100  mg of feces, using the Zymo Fecal DNA Miniprep Kit according to the manufacturer’s instructions (Zymo Research, Irvine, CA). Negative controls to detect kit contamination were included and failed to produce visible PCR bands in an agarose gel but were analyzed as quality controls. Samples were subjected to 16S ribosomal RNA (rRNA) gene sequencing as previously described using the 515F primer (5′-GTGYCAGCMGCCGCGGTAA-3′) and 806R primer (5′-GGACTACNVGGGTWTCTAAT-3′) with appended barcodes and sequencing adapters (19). Quantification of the total *B. infantis* was performed by quantitative real-time PCR using Blon_2348 sialidase gene primers Inf2348F (5′-ATA CAG CAG AAC CTT GGC CT-3′), Inf2348_R (5′-GCG ATC ACA TGG ACG AGA AC-3′), and Inf2348_P (5′-/56-FAM/TTT CAC GGA /ZEN/TCA CCG GAC CAT ACG/3lABkFQ/−3′). The *Blon_2348* gene is found in all *B. infantis* strains, including EVC001. The primer and probe sequence specificity has been previously described (27). Each reaction contained 10 µL of 2× TaqMan Universal Master Mix II with UNG master mix (Thermo Fisher Scientific, Waltham, MA), 0.9 µM of each primer, 0.25 µM probe, and 5 µL of template DNA. Thermal cycling was performed on a QuantStudio 3 Real-Time PCR System (Thermo Fisher Scientific, Waltham, MA) and consisted of an initial UNG activation step of 2 min at 50°C, followed by a 10 min denaturation at 95°C, succeeded by 40 cycles of 15 s at 95°C and 1 min at 60°C. All samples were run in duplicate with a standard curve on each plate. Quantification of *B. infantis* was determined (CFU/g stool) using a standard curve of genomic DNA derived from a pure culture of *B. infantis* EVC001 using CFU counts and normalized for input stool wet weight (28). Standard curve genomic DNA was extracted from 1 mL aliquots of *B. infantis* EVC001 grown anaerobically at 37°C for 16 h in deMann Rogosa Sharpe (MRS) medium (BD Biosciences, San Jose, CA) supplemented with 0.05% L-cysteine HCl. CFU counts of the 16 h *B. infantis* EVC001 culture were determined by serial dilution in 0.9% NaCl on MRS agar plates containing 0.05% L-cysteine HCl. Plates were incubated anaerobically at 37°C for 48 h, then counted, and the CFU/mL value was calculated.

### 16S rRNA sequencing Analysis

Raw sequencing files were analyzed using QIIME 2 (29). 16S rRNA amplicon sequencing yielded an average of 7,713 reads per sample (±3.648 SD). The reads were processed first using DADA2, which involved quality trimming, joining, and denoising before identifying amplicon sequence variants (ASVs) (30). Taxonomic identification of ASVs was completed using a naïve Bayesian classifier (31) trained on the Silva v138 database (32), and ASV sequences were aligned using FastTree to generate a phylogenetic tree for ASVs (33). Alpha and beta diversity indices were calculated using frequency tables rarefied to 1,179 reads per sample, which was chosen to maintain 90% or more samples within the data set and an alpha rarefaction confirmed that sequencing depth across samples had plateaued at this depth. The Shannon diversity of each sample before day 7 (baseline), day 35 (end of supplementation period), and day 63 (after supplementation) was calculated using QIIME2 and differences in the Shannon index between groups were conducted using a nonparametric Wilcoxon test. The weighted UniFrac distance matrix was chosen for analysis, and the significance of treatment on beta diversity was determined using the Adonis test (34).

Multivariate association with linear models 2 (MaAsLin2) (35), which employs generalized linear models (GLMs), was run via a linear analysis method using a false-discovery rate (FDR) of 0.05, and a minimum prevalence of 10% of samples. Fixed effects used in the MaAsLin2 model included treatment (placebo and combined low, medium, and high *B. infantis* doses), delivery mode (cesarean or vaginal delivery), and timing of before, during, or after supplementation periods. Participant ID was used as a random variable. *P*-values were adjusted via FDR (Q-values) and considered significant if Q-value <0.05. Taxa presented in this analysis were present at greater than 0.1% mean relative abundance across all samples. Raw sequencing data are publicly deposited in the NCBI SRA under BioProjectID accession number PRJNA1188647.

### Statistics

The primary endpoint was analyzed using the intent-to-treat population, which includes all participants who were randomized. All other secondary analyses were conducted using the per-protocol population, which includes all randomized participants who did not have major protocol deviations and is considered a supportive population for the primary and key secondary efficacy analyses. The levels of infant fecal *B. infantis* and total fecal *Bifidobacterium* were log_10_-transformed prior to analysis. All hypothesis testing for primary and secondary analyses, excluding 16S rRNA data, was two-sided, and the family-wise type I error rate (α) was maintained at 0.05. For some statistical analyses, the low-, medium-, and high-dose groups were combined, and this group is referred to as EVC throughout.

### Primary analysis

The primary analysis utilized the non-parametric Kruskal-Wallis test to compare distributions of infant fecal *B. infantis* (as measured by *B. infantis* qPCR) at day 28 in all treatment groups. If the *P*-value for the test of treatment (all doses) was statistically significant at the α = 0.05 level, the sequential Holm’s step-down testing strategy was employed to determine which dose(s) of *B. infantis* were statistically significantly more effective than placebo for increasing levels of infant fecal *B. infantis* following 28 days of supplementation. Pairwise comparisons of each dose of *B. infantis* versus placebo were conducted using the nonparametric Wilcoxon rank sum test.

### Secondary analyses

The difference among the three *B. infantis* doses on levels of infant fecal *B. infantis* on day 28 was the key secondary efficacy analysis. All hypothesis testing was two-sided, and the family-wise type I error rate (α) for the key secondary efficacy analysis was maintained at 0.05 through application of the Holm’s procedure. All three doses of *B. infantis* were required to be statistically superior to placebo to formally test all pairwise comparisons of *B. infantis* doses. Pairwise comparisons of each dose of *B. infantis* were conducted using the Wilcoxon Rank Sum test. Holm’s procedure was applied to control the family-wise type I error rate (α) to 0.05 for multiple treatment comparisons, and *P* values were adjusted based on three comparisons (α/3, α/2, and α) for placebo vs. each dose and each dose vs. another dose.

Differences in fecal *B. infantis* levels among treatment groups at baseline were determined using Kruskal-Wallis tests. Differences in fecal *B. infantis* and total *Bifidobacterium* levels at post-baseline time points (days 10, 14, 21, 28, 35, 42, and 63) between treatment groups were determined using a rank-based one-way ANOVA model (van der Waerden normal scores), a non-parametric ANOVA appropriate for addressing multiple time points. Pairwise comparisons of each *B. infantis* dose versus placebo were conducted using the Wilcoxon rank sum test; unadjusted and Dunnett adjusted *P*-values were generated.

As a sensitivity analysis, estimates of treatment effect at day 28 and across all post-baseline time points based on missing at random assumption (MAR) for missing values were obtained from a mixed model for repeated measurements (MMRM), inclusive of values from all post-baseline sampling time points and with baseline as a covariate and factors for treatment, time, and interaction of treatment by time. Due to concerns about the ability to satisfy the normality assumption, MMRM modeled the van der Waerden normal score transformed ranks of infant fecal *B. infantis* levels rather than actual values. Specifically, ranks were determined separately for all time points included, including baseline, followed by van der Waerden normal score derived from the rank.

Differences in infant and maternal baseline characteristics; infant weight (baseline, supplementation, and post-supplementation); sleep and crying hours at baseline, during the supplementation period, and the change between the baseline and supplementation period; and stool number, size, and consistency (baseline, supplementation, and post-supplementation) between all four treatment groups (placebo, low, medium, and high) were determined using Kruskal-Wallis and Wilcoxon rank sum tests (placebo vs. all *B. infantis* doses combined). Pairwise comparisons were conducted using the Wilcoxon rank sum test. Differences in the number of infant stools in each treatment group: placebo, low, medium, high, and EVC (all doses) for matched samples across time points were determined using the Friedman test.

Comparisons of the proportion of days infants in the placebo versus EVC group experienced any of the following symptoms or conditions: cold, runny nose, or cough; fever at or above 103°F blood in stool; prolonged abdominal bloating or straining; vomiting; and diaper rash were conducted using Wilcoxon rank sum test for the supplementation and post-supplementation period. These data were binned across the three study periods: baseline, supplementation, and post-supplementation, and means and proportions were calculated for continuous and categorical variables across each study period. Proportions for binary categorical variables were calculated as the number of days reported/total number of days in each study period. The calculated values were multiplied by 100 to generate percentages.

## RESULTS

### Study participants

**Fig 1:**
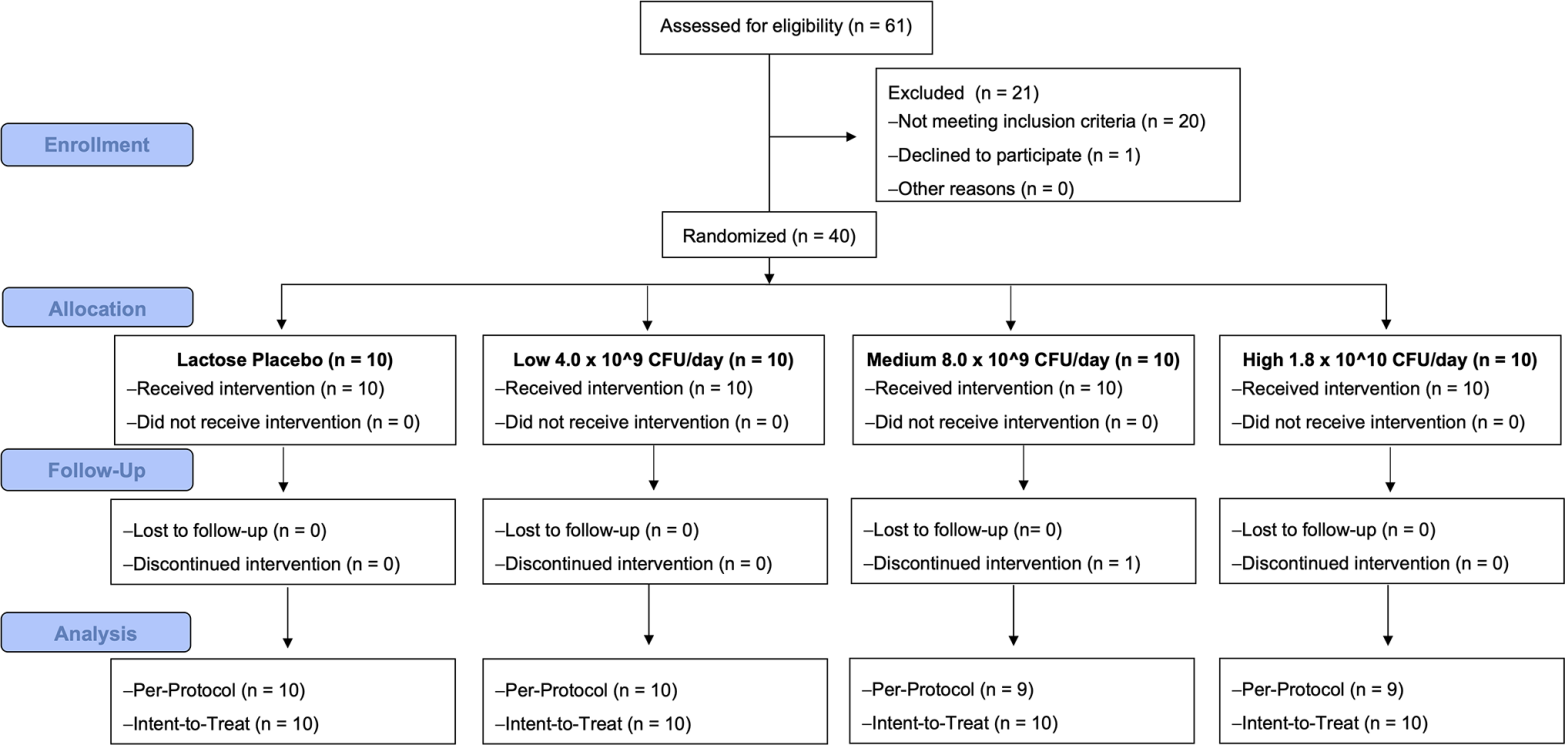
Consort diagram describing the number of participants screened, enrolled, randomized, or discontinued throughout the study period, and the number of participants included in the per-protocol and intent-to-treat analyses.

Sixty-one mothers were recruited, screened for eligibility to participate in the study between April 2018 and November 2018. Forty-one women met initial study criteria and were enrolled in the study, of which 40 were randomly assigned into one of four treatment groups, as one participant withdrew from the study prior to randomization (Fig. 1). Participants completed the trial in March 2019. Data for all randomized participants in each group (*n* = 10 per group) are reported, except for one participant in the medium-dose group (8.0 × 10^9^ CFU/day) who withdrew during the supplementation period and for which there are no data reported during the supplementation or post-supplementation phases. Additionally, one participant in the high-dose group reported administering antibiotics to her infant during the supplementation period and was excluded from the primary analysis. The overall attrition rate for this study was 5%, consistent with our previous probiotic study in healthy, breastfed, term infants (36). In total, 97.5% of randomized participants consumed at least 21 once-daily servings of *B. infantis*. No infants consumed more than one dose per day.

### Maternal characteristics

Maternal age at enrollment, pre-pregnancy BMI, weight gain during pregnancy, number of times pregnant, number of live births, number of children living in the home, and number of hours in labor (Table 1) were not significantly different between treatment groups. Additional maternal baseline characteristics can be found in Table S1.

**TABLE 1:** Maternal baseline characteristics

Parameter	Placebo (*n* = 10)		4B CFU/day (*n* = 10)		8B CFU/day (*n* = 10)		18B CFU/day (*n* = 10)		EVC^*a*^ (*n* = 30)	
Age at enrollment, yrs (mean, SD)	33.2, 1.9		33.0, 4.7		34.3, 3.5		32.1, 4.7		33.1, 4.3	
Ethnicity, *n* (%)										
Not Hispanic	9 (90%)		9 (90%)		10 (100%)		9 (90%)		28 (93.3%)	
Hispanic	1 (10%)		1 (10%)		0 (0%)		0 (0%)		1 (3.3%)	
Unsure	0 (0%)		0 (0%)		0 (0%)		1 (10%)		1 (3.3%)	
Race, *n* (%)										
Asian	1 (10%)		1 (10%)		2 (20%)		0 (0%)		3 (10%)	
Black or African American	0 (0%)		0 (0%)		0 (0%)		2 (20%)		2 (6.7%)	
White (including Middle Eastern)	8 (80%)		8 (80%)		6 (60%)		8 (80%)		22 (73.3%)	
Two or more races	1 (10%)		1 (10%)		2 (20%)		0 (0%)		3 (10%)	
Education, *n* (%)										
Some college, no degree	0 (0%)		1 (10%)		0 (0%)		2 (20%)		3 (10%)	
Associate degree	1 (10%)		0 (0%)		0 (0%)		1 (10%)		1 (3.3%)	
Bachelor's degree (BA, BS, etc.)	5 (50%)		4 (40%)		4 (40%)		5 (50%)		13 (43.3%)	
Secondary degree (MA, MS, MEng, MSW, etc.)	2 (20%)		5 (50%)		4 (40%)		0 (0%)		9 (30%)	
Professional or doctorate (MD, DDS, JD, PhD, EdD, etc.)	2 (20%)		0 (0%)		2 (20%)		2 (20%)		4 (13.3%)	
Marital status, *n* (%)										
Married/couple	10 (100%)		9 (90%)		10 (100%)		10 (100%)		29 (96.7%)	
Never married	0 (0%)		1 (10%)		0 (0%)		0 (0%)		1 (3.3%)	
Pre-pregnancy BMI (mean, SD)	26.3, 4.4		25.1, 4.8		24.9, 5.1		26.7, 5.0		25.6, 4.8	
Pregnancy weight gain (kg [mean, SD])	29.8, 8.8		27.4, 16.7		26.1, 9.2		26.0, 12.5		26.5, 12.7	
Hours in labor (h [mean, SD])	15.2, 15.0		13.2, 13.2		16.6, 8.0		14.5, 11.2		14.8, 10.7	
Number of pregnancies (mean, SD)	1.6, 1.3		1.2, 1.4		1.3, 1.8		1.7, 1.4		1.4, 1.5	
Number of live births (mean, SD)	1.6, 0.7		1.2, 0.4		1.0, 0.0		1.3, 1.0		1.2, 0.6	
Number of children (mean, SD)	1.3, 0.9		0.8, 0.6		0.6, 0.5		0.9, 1.0		0.8, 0.7	
Parity, *n* (%)										
Primiparous	2 (20%)		4 (40%)		4 (40%)		3 (30%)		11 (36.7%)	
Multiparous	8 (80%)		6 (60%)		6 (60%)		7 (70%)		19 (63.3%)	
Delivery location, *n* (%)										
Hospital or birthing center	8 (80%)		10 (100%)		10 (100%)		10 (100%)		30 (100%)	
Home birth	2 (20%)		0 (0%)		0 (0%)		0 (0%)		0 (0%)	
Mode of delivery, *n* (%)										
Vaginal	6 (60%)		7 (70%)		6 (60%)		8 (80%)		21 (70%)	
Vaginal water birth	2 (20%)		1 (10%)		2 (20%)		0 (0%)		3 (10%)	
C-section, elective	0 (0%)		2 (20%)		2 (20%)		2 (20%)		6 (20%)	
C-section, emergent	2 (20%)		0 (0%)		0 (0%)		0 (0%)		0 (0%)	

^*a*^ EVC, the mean across all EVC001 doses.

### Infant characteristics

Infant age at enrollment, birth weight, birth length, and gender were not significantly different between treatment groups (Table 2). Infant gestational age at birth was significantly different between the placebo, low-, medium-, and high-dose groups (*P* < 0.05) and between placebo and the EVC group (*P* < 0.05). Pairwise comparisons showed that there was a significant difference between the placebo group (mean = 40.2 weeks) and the low-dose group (mean = 38.9 weeks) (*P* < 0.05). Differences in gestational age at birth are not biologically significant as all infants in this study were born full-term. Infant weight was not significantly different between treatment groups at study days 7, 37, or 65 (Fig. S1). Additional infant baseline characteristics can be found in Table S2.

**TABLE 2:** Infant baseline characteristics

Infant baseline characteristics	Placebo (*n* = 10)		4B CFU/day (*n* = 10)		8B CFU/day (*n* = 10)		18B CFU/day (*n* = 10)		EVC^*a*^ (*n* = 30)	
	Mean	SD	Mean	SD	Mean	SD	Mean	SD	Mean	SD
Gestational age (wks)	40.2^*c*^	1.1	38.9	1.0	39.8	0.6	39.9	1.2	39.5	1.0
Birth weight (g)	3,454.4	410.0	3,477.1	536.2	3,468.6	394.7	3,647.2	356.4	3,530.9	428.9
Infant birth length (cm)^*b*^	50.1	2.9	51.2	1.6	50.4	1.8	50.7	3.2	50.8	2.2
Age at enrollment (days)	95.8	18.5	87.7	23.5	95.5	19.3	101.1	13.0	94.8	19.2
Infant gender, *n* (%)										
Male	3 (30%)		4 (40%)		5 (50%)		3 (30%)		12 (40%)	
Female	7 (70%)		6 (60%)		5 (50%)		7 (70%)		18 (60%)	
Ethnicity, *n* (%)										
Not Hispanic	9 (90%)		9 (90%)		9 (90%)		8 (80%)		26 (86.7%)	
Hispanic	1 (10%)		1 (10%)		1 (10%)		1 (10%)		3 (10%)	
Unsure	0 (0%)		0 (0%)		0 (0%)		1 (10%)		1 (3.3%)	
Race, *n* (%)										
Asian	0 (0%)		1 (10%)		2 (20%)		0 (0%)		3 (10%)	
Black or African American	0 (0%)		0 (0%)		0 (0%)		0 (0%)		0 (0%)	
White (including Middle Eastern)	9 (90%)		7 (70%)		6 (60%)		6 (60%)		19 (63.3%)	
Two or more races	1 (10%)		2 (20%)		2 (20%)		3 (30%)		7 (23.3%)	
Refuse	0 (0%)		0 (0%)		0 (0%)		1 (10%)		1 (3.3%)	

^*a*^ EVC, the mean across all EVC001 doses.

^*b*^ *n* = 9, one participant in the placebo group did not report birth length.

^*c*^ Significant differences between placebo, low, medium, and high doses, and between placebo and EVC, *P *< 0.05 (Kruskal-Wallis). Pairwise comparisons showed a significant difference between the placebo and low dose, *P* < 0.05 (Wilcoxon rank sum).

### Infant diet

All women reported feeding their infant breast milk (at the breast or by bottle), and no one reported feeding their infant any amount of infant formula or non-study probiotics throughout the duration of the study (Table S3). Two women in the high-dose group reported feeding their infants solid food during the post-supplementation period. One mother in the high-dose group reported feeding her infant antibiotics during the supplementation and post-supplementation periods to treat her infant’s eczema that was diagnosed prior to enrollment in the study.

### Infant gastrointestinal health and tolerability

**Fig 2:**
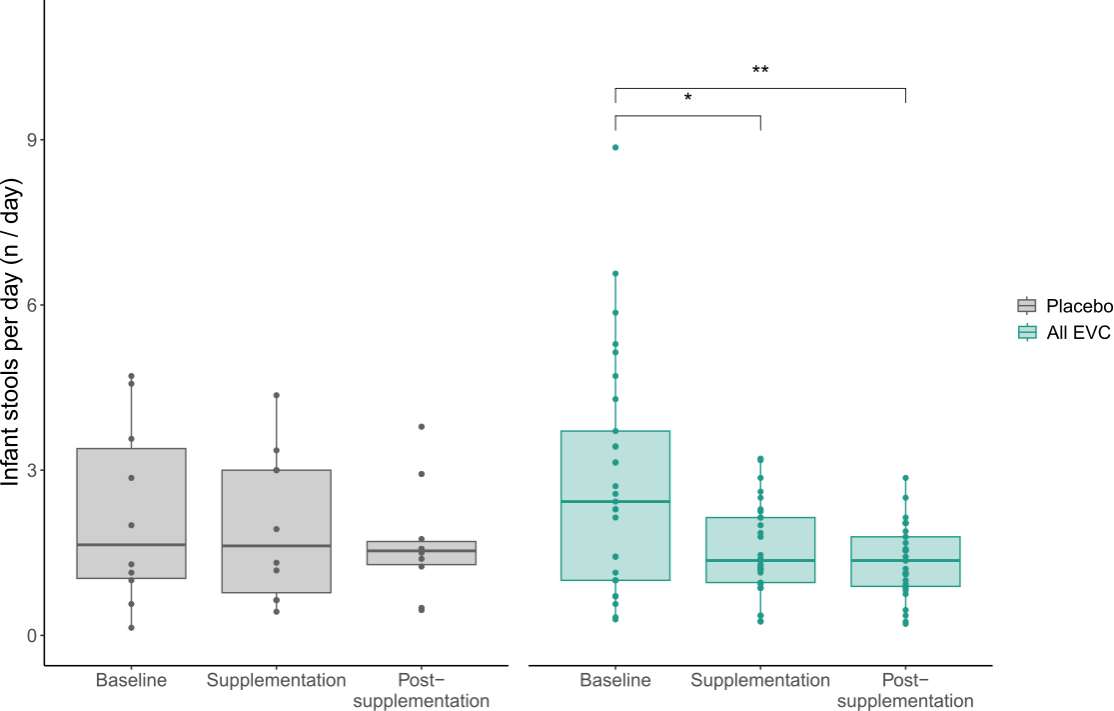
The number of stools per day across time and among placebo and EVC (all doses combined). Bonferroni adjusted *P* < 0.0001 (****) for differences in the EVC group (Friedman test) with Bonferroni-adjusted Wilcoxon rank-sum test comparisons between baseline and during or after supplementation (*P* < 0.05, *; *P* < 0.01, **).

The number of infant stools was not significantly different between treatment groups during baseline, supplementation, and post-supplementation. The number of infant stools was significantly different within the low-dose group only across time (*P* < 0.001) between the baseline and supplementation (*P* < 0.05) and the baseline and post-supplementation (*P* < 0.01); however, after Bonferroni correction, only baseline and post-supplementation were statistically different from each other within the low-dose group (*P* < 0.01). The number of infant stools was significantly different within the EVC group across time (*P* < 0.0005) between the baseline and supplementation time points (*P* < 0.0005), baseline and post-supplementation time points (*P* < 0.0005), and supplementation and post-supplementation time points (*P* < 0.0005). After Bonferroni correction, only the baseline and supplementation (*P* < 0.001) and baseline and post-supplementation time points (*P* < 0.0005) were significantly different from each other within the EVC group (Fig. 2; Fig. S2; Table S3).

Maternal reports of stool consistency as the proportion of watery, formed, soft, or hard stools during the baseline, supplementation, and post-supplementation periods were not significantly different between treatment groups (Fig. S3; Table S3). Maternal reports for the size of stools as the proportion of stools that were a smear on the infant’s diaper, measured up to 25% of the diaper, 25%–50% of the diaper, or >50% of the diaper during the baseline, supplementation, and post-supplementation periods were not significantly different between treatment groups (Fig. S4; Table S3).

The proportion of infants who experienced a cold, runny nose, or cough, fever at or above 103°F, blood in stool, prolonged abdominal bloating or straining, vomiting, and diaper rash was not significantly different between the placebo and EVC group at any time period. (Table S3).

### Infant sleep and crying

Maternal reports for the hours their infant spent sleeping or crying/fussing were not significantly different between treatment groups at baseline or during the supplementation period. The change in hours spent sleeping or crying/fussing from baseline was not significantly different between treatment groups (Table S4).

### Changes to the fecal microbiome

**Fig 3:**
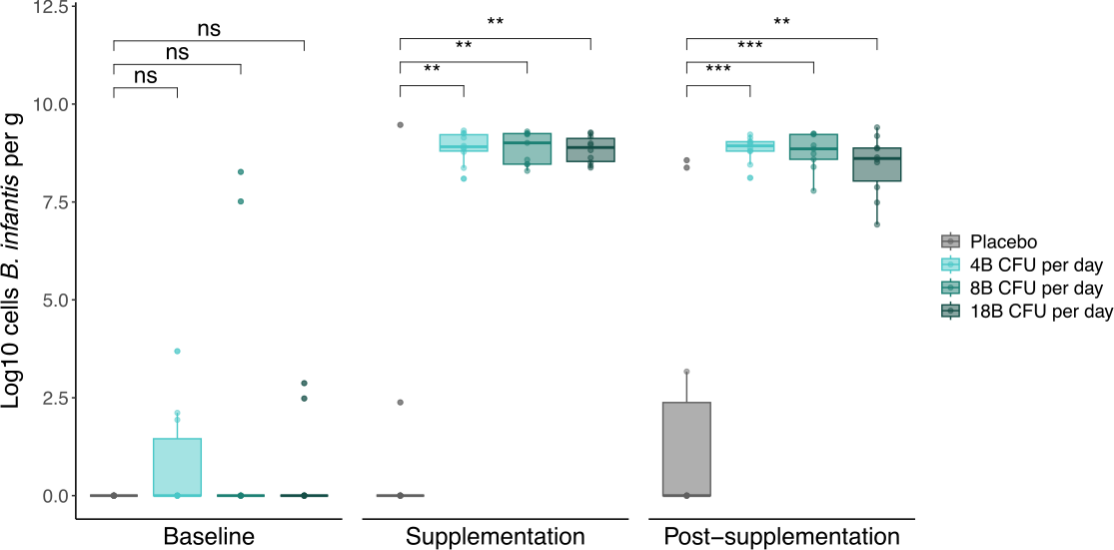
Colony-forming units (CFU) of infant fecal *B. infantis* measured by qPCR at baseline, supplementation, and post-supplementation periods between treatment groups. ns = not significant, ** *P* < 0.01, *** *P* < 0.0001 for differences between treatment groups at baseline (before day 7), supplementation (day 28), and post-supplementation (day 63), determined using a Wilcoxon rank-sum test. Differences at baseline and post-supplementation were determined using Kruskal-Wallis and pairwise comparisons during supplementation showed that each dose was significantly different from placebo (*P* < 0.01). Differences at post-supplementation were determined by an ANOVA model, and pairwise comparisons showed that each dose was significantly different from placebo (*P* < 0.0002).

All stool samples arrived at the study site frozen, and no samples had to be re-collected. There was a significant difference in fecal *B. infantis* levels at day 28 between the treatment groups (all doses) and placebo (*P* < 0.01) (Fig. 3). Fecal *B. infantis* levels in the low-, medium-, and high-dose groups were significantly different from placebo at day 28 (Holm’s adjusted: low: *P* < 0.01, medium *P* < 0.01, high *P* < 0.01). There was no significant difference between fecal *B. infantis* levels, as assessed by qPCR, between treatment groups at baseline or thereafter (Holm’s adjusted *P* > 0.05). The MMRM confirmed these findings (*P =* 0.001–0.01). There were significant differences in the ranks of fecal *B. infantis* levels between treatment groups (all doses) and placebo at day 10 (*P* < 0.0005), day 14 (*P* < 0.0005), day 21 (*P* < 0.0005), day 35 (*P* < 0.0005), day 42 (*P* < 0.0005), and day 63 (*P* < 0.0005) (Fig. S5). Pairwise comparisons identified significant differences in fecal *B. infantis* levels after Dunnett adjustment between the low, medium, and high doses compared with placebo at all post-baseline time points (low: *P* < 0.0005, medium *P* < 0.0005, high *P* < 0.0005). The MMRM confirmed these findings (*P* < 0.0001).

There were significant differences in the ranks of fecal *Bifidobacterium* levels measured by qPCR between treatment groups at day 10 (*P* < 0.01), day 14 (*P* < 0.01), day 21 (*P* < 0.05), day 28 (*P* < 0.05), day 35 (*P* < 0.001), and day 42 (*P* < 0.0005) (Fig. S6). After Dunnett adjustment, pairwise comparisons showed that at day 63, there was no significant difference in fecal *Bifidobacterium* levels between treatment groups. At day 10, there were significant differences between the low-dose (*P* < 0.01) and the high-dose (*P* < 0.01) when compared with placebo. At day 14, there were significant differences between the low (*P* < 0.01), medium (*P* < 0.01), and high (*P* < 0.01) doses when compared with placebo. At day 21, there were significant differences between the medium (*P* < 0.05) and high (*P* < 0.05) doses when compared with placebo. At day 28, there was a significant difference between the medium (*P* < 0.05) dose and placebo. At day 35, there were significant differences between the low (*P* < 0.05), medium (*P* < 0.001), and high (*P* < 0.01) doses when compared with placebo. At day 42, there were significant differences between the low (*P* < 0.0005) and medium (*P* < 0.01) doses when compared with placebo. The MMRM confirmed these findings (*P =* 0.0001–0.01).

**Fig 4:**
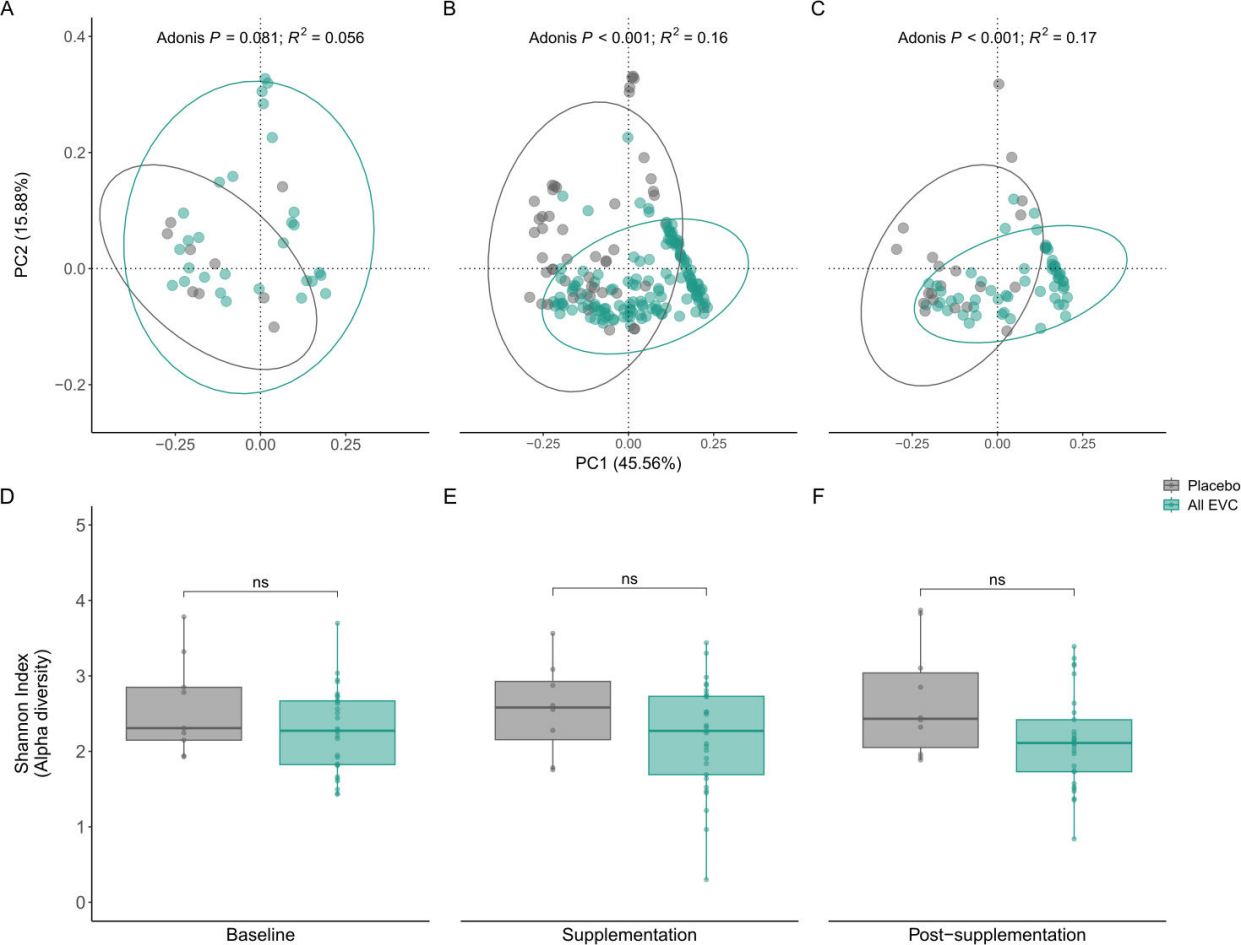
Beta diversity of all gut microbiome samples at (**A**) baseline, (**B**) supplementation, and (**C**) post-supplementation periods, as determined using the Weighted UniFrac index and visualized across the top two principal components explaining 57.6% of the variation observed. EVC001 treatment or placebo control has a significant association with beta diversity as determined by Adonis test. Shannon diversity index of all gut microbiome samples at (**D**) baseline, (**E**) supplementation, and (**F**) post-supplementation periods. Statistical differences were determined using the Wilcoxon rank-sum test. *P* < 0.05. Not statistically significant (ns).

To compare the gut microbiome composition before, during, and after supplementation with *B. infantis,* a weighted UniFrac distance metric was applied to the samples. Although the communities were not significantly different at baseline (Adonis *P* > 0.05), supplementation with *B. infantis* significantly altered the microbiome composition (Adonis *P* < 0.001; Fig. 4A through C). In terms of alpha diversity, the Shannon diversity index was not significantly different between samples before, during, or after supplementation, indicating that there was no measurable impact on the richness and evenness of the community among these samples (Fig. 4D through F).

**Fig 5:**
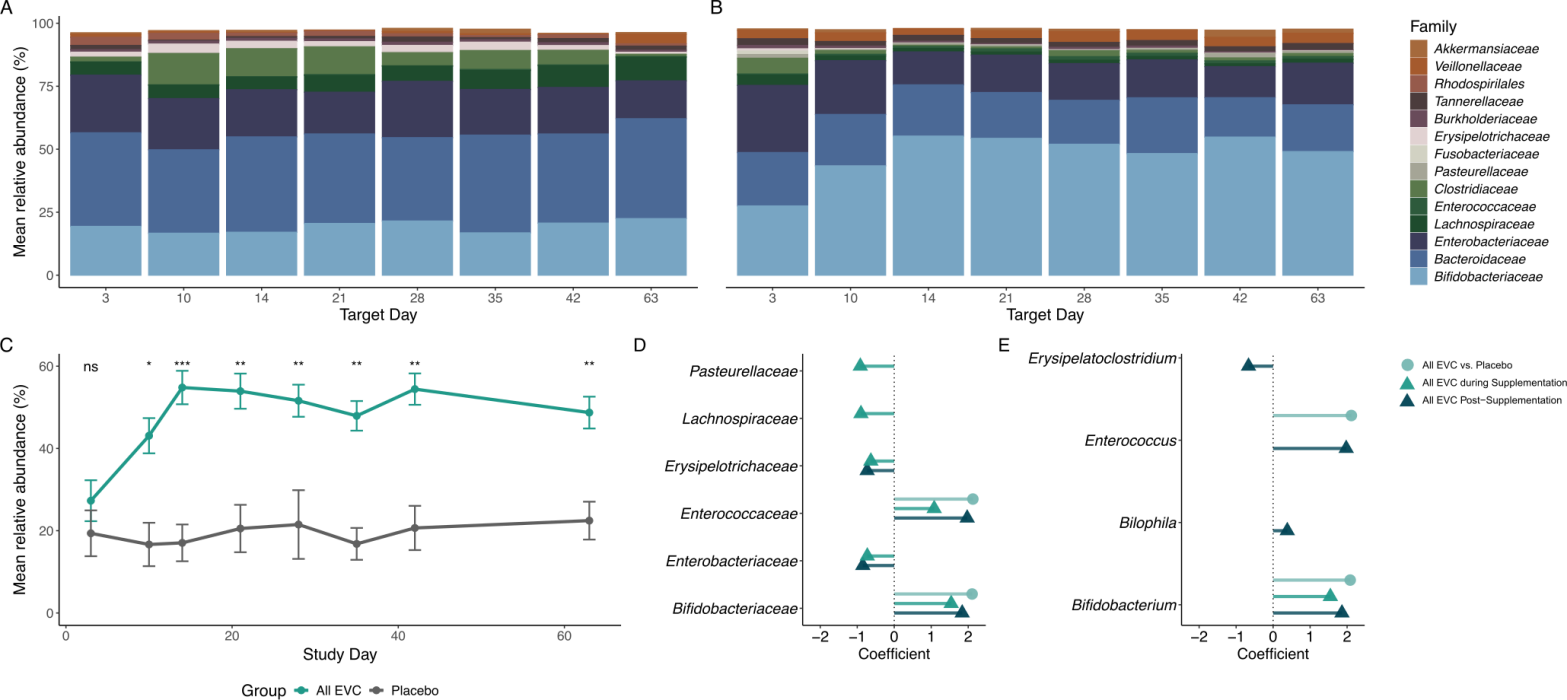
Mean relative abundance (%) of bacterial families present above 0.1% mean relative abundance among the (**A**) placebo group and (**B**) all EVC (all doses combined) group across all time points. (**C**) The mean relative abundance of *Bifidobacteriaceae* is shown (±SEM). Multivariate modeling with MaAsLin2 of taxa at the (**D**) family and (**E**) genus levels. Circles indicate comparisons between the All EVC-supplemented group with the placebo; triangles indicate comparisons between the All EVC-supplemented group during or post-supplementation, relative to baseline. Positive coefficients indicate a relative increase, whereas the negative coefficients indicate a relative decrease.

We used multivariate linear modeling with MaAsLin2 to determine if *B. infantis* supplementation altered gut microbial taxa, as measured by 16S rRNA sequencing (Fig. 5). Although mean *Bifidobacteriaceae* levels in the placebo group were ~20% of the community throughout the duration of the study, *Bifidobacteriaceae* levels in the EVC (all doses combined) group were above 50% after baseline (Fig. 5; Table 3), which was reflected in the multivariate modeling results. After controlling for delivery mode (cesarean section or vaginal delivery), and time period (before, during, or after *B. infantis* supplementation), families with greater than 0.1% abundance that were significantly more abundant among infants in the EVC group included *Bifidobacteriaceae* and *Enterococcaceae* (*q* < 0.05), whereas *Enterobacteriaceae, Erysipelotrichaceae*, *Lachnospiraceae*, and *Pasteurellaceae* were all significantly less abundant (*q* < 0.05; Fig. 5D, Table 3). Among genera present at greater than 0.1%, *Bifidobacterium* was significantly more abundant among infants in the EVC group during supplementation and post-supplementation, relative to the baseline period, whereas *Enterococcus* was more abundant among infants in the EVC group post-supplementation, relative to the baseline period (*q* < 0.05; Fig. 5E, Table 3). No bacterial families or genera present at greater than 0.1% mean relative abundance across the samples were significantly impacted by the delivery mode (*q* > 0.05).

**TABLE 3:** Mean relative abundance (± SD) of bacterial families in infant fecal samples as measured by 16s rRNA amplicon sequencing

Study day	Family	Placebo (*n* = 10)	4B CFU/day (*n* = 10)	8B CFU/day (*n* = 10)	18B CFU/day (*n* = 10)	EVC^*a*^ (*n* = 30)
3	*Akkermansiaceae*	0.56 ± 1.78	0.00 ± 0.00	1.77 ± 5.30	0.00 ± 0.00	0.55 ± 2.95
	*Bacteroidaceae*	37.16 ± 26.35	23.47 ± 30.71	18.73 ± 22.15	20.69 ± 24.92	21.04 ± 25.43
	*Bifidobacteriaceae*	19.37 ± 17.62	28.71 ± 30.34	20.77 ± 28.65	31.74 ± 22.82	27.29 ± 26.82
	*Burkholderiaceae*	1.01 ± 3.03	0.00 ± 0.00	0.66 ± 1.98	3.26 ± 6.55	1.33 ± 4.12
	*Clostridiaceae*	1.79 ± 2.76	3.56 ± 4.37	11.30 ± 21.07	4.26 ± 11.49	6.20 ± 13.70
	*Enterobacteriaceae*	22.88 ± 19.97	26.25 ± 15.02	29.60 ± 23.86	23.83 ± 16.69	26.45 ± 18.17
	*Enterococcaceae*	0.16 ± 0.20	0.20 ± 0.35	0.17 ± 0.26	0.80 ± 1.56	0.40 ± 0.96
	*Erysipelotrichaceae*	1.83 ± 3.47	0.75 ± 1.41	0.22 ± 0.53	1.54 ± 3.23	0.86 ± 2.09
	*Lachnospiraceae*	5.12 ± 7.24	3.52 ± 4.81	3.27 ± 4.94	5.43 ± 10.57	4.10 ± 7.16
	*Pasteurellaceae*	0.21 ± 0.44	2.39 ± 4.48	1.01 ± 2.67	0.93 ± 2.25	1.46 ± 3.25
	*Streptococcaceae*	0.23 ± 0.39	0.30 ± 0.49	1.82 ± 3.34	0.35 ± 0.53	0.79 ± 1.96
	*Tannerellaceae*	1.67 ± 4.38	4.87 ± 14.07	2.31 ± 6.17	0.67 ± 1.62	2.63 ± 8.86
	*Veillonellaceae*	1.23 ± 1.93	5.21 ± 8.60	1.20 ± 1.40	3.42 ± 5.17	3.35 ± 5.97
10	*Akkermansiaceae*	0.44 ± 1.39	0.00 ± 0.00	4.01 ± 12.03	0.00 ± 0.00	1.24 ± 6.70
	*Bacteroidaceae*	33.11 ± 24.14	18.34 ± 24.54	21.76 ± 23.73	20.79 ± 20.34	20.25 ± 22.12
	*Bifidobacteriaceae*	16.66 ± 16.68	43.10 ± 27.24	38.45 ± 24.49	47.25 ± 18.29	43.09 ± 23.03
	*Burkholderiaceae*	1.20 ± 3.73	0.00 ± 0.00	0.93 ± 2.80	2.03 ± 3.54	0.99 ± 2.65
	*Clostridiaceae*	12.35 ± 22.50	3.21 ± 7.25	0.68 ± 1.35	0.42 ± 0.96	1.46 ± 4.40
	*Enterobacteriaceae*	20.33 ± 18.17	20.52 ± 15.34	25.28 ± 26.52	18.21 ± 19.12	21.20 ± 20.07
	*Enterococcaceae*	0.17 ± 0.24	0.45 ± 0.69	0.75 ± 1.65	0.78 ± 1.07	0.66 ± 1.15
	*Erysipelotrichaceae*	3.60 ± 7.82	0.42 ± 0.91	0.01 ± 0.02	0.73 ± 2.19	0.40 ± 1.38
	*Lachnospiraceae*	5.31 ± 7.93	2.68 ± 5.42	1.27 ± 1.53	1.58 ± 2.24	1.86 ± 3.48
	*Pasteurellaceae*	0.22 ± 0.45	0.34 ± 0.44	0.31 ± 0.50	0.43 ± 1.30	0.36 ± 0.83
	*Streptococcaceae*	0.20 ± 0.33	2.16 ± 5.79	0.54 ± 0.60	0.98 ± 1.78	1.25 ± 3.52
	*Tannerellaceae*	0.36 ± 0.85	3.41 ± 8.29	0.72 ± 1.47	1.17 ± 3.52	1.80 ± 5.31
	*Veillonellaceae*	0.68 ± 0.92	3.33 ± 6.49	3.81 ± 9.36	3.10 ± 5.52	3.40 ± 6.96
14	*Akkermansiaceae*	0.77 ± 2.44	0.00 ± 0.00	0.00 ± 0.00	0.00 ± 0.00	0.00 ± 0.00
	*Bacteroidaceae*	37.85 ± 25.29	17.43 ± 27.09	15.93 ± 16.48	26.40 ± 27.56	20.20 ± 24.31
	*Bifidobacteriaceae*	17.05 ± 14.13	52.63 ± 22.04	62.87 ± 19.58	50.53 ± 22.80	54.81 ± 21.50
	Burkholderiaceae	1.18 ± 3.70	0.02 ± 0.06	0.68 ± 1.93	1.38 ± 2.33	0.70 ± 1.77
	Clostridiaceae	11.11 ± 19.96	0.91 ± 1.56	0.35 ± 0.66	0.12 ± 0.23	0.46 ± 1.03
	*Enterobacteriaceae*	18.71 ± 13.82	16.37 ± 10.49	10.49 ± 6.75	11.54 ± 9.80	12.96 ± 9.35
	*Enterococcaceae*	0.06 ± 0.11	0.45 ± 0.88	1.09 ± 1.47	0.95 ± 0.74	0.81 ± 1.04
	*Erysipelotrichaceae*	2.89 ± 6.99	0.15 ± 0.42	0.15 ± 0.32	0.08 ± 0.11	0.12 ± 0.30
	*Lachnospiraceae*	5.06 ± 11.18	1.22 ± 2.10	1.66 ± 2.48	1.22 ± 1.99	1.35 ± 2.11
	*Pasteurellaceae*	0.12 ± 0.28	1.04 ± 1.51	0.35 ± 0.96	0.81 ± 1.50	0.76 ± 1.35
	*Streptococcaceae*	0.52 ± 0.92	1.29 ± 1.96	0.74 ± 0.73	0.85 ± 1.34	0.98 ± 1.44
	*Tannerellaceae*	0.67 ± 1.42	2.95 ± 8.90	2.07 ± 3.84	1.84 ± 4.25	2.30 ± 6.04
	*Veillonellaceae*	0.35 ± 0.42	2.95 ± 4.68	2.82 ± 4.87	2.29 ± 1.88	2.68 ± 3.84
21	*Akkermansiaceae*	0.08 ± 0.27	0.00 ± 0.00	2.98 ± 8.93	0.00 ± 0.00	0.92 ± 4.98
	*Bacteroidaceae*	35.54 ± 25.05	16.00 ± 23.63	16.34 ± 19.59	21.67 ± 20.71	18.06 ± 20.83
	*Bifidobacteriaceae*	20.53 ± 18.24	50.00 ± 26.76	57.73 ± 22.22	54.42 ± 21.47	53.92 ± 23.01
	*Burkholderiaceae*	1.08 ± 3.33	0.00 ± 0.00	0.37 ± 1.11	1.19 ± 2.61	0.53 ± 1.68
	*Clostridiaceae*	10.91 ± 23.25	0.95 ± 1.59	0.49 ± 1.11	0.75 ± 1.36	0.74 ± 1.34
	*Enterobacteriaceae*	16.56 ± 8.63	19.59 ± 17.96	12.76 ± 8.43	11.63 ± 5.74	14.72 ± 12.15
	*Enterococcaceae*	0.35 ± 0.79	0.40 ± 0.68	2.41 ± 3.75	1.13 ± 1.45	1.28 ± 2.35
	*Erysipelotrichaceae*	1.97 ± 4.56	0.23 ± 0.65	0.08 ± 0.17	0.03 ± 0.06	0.11 ± 0.39
	*Lachnospiraceae*	6.65 ± 10.17	1.78 ± 3.82	0.76 ± 1.16	1.07 ± 1.49	1.22 ± 2.45
	*Pasteurellaceae*	0.19 ± 0.43	0.46 ± 0.96	0.06 ± 0.09	1.39 ± 2.28	0.66 ± 1.51
	*Streptococcaceae*	0.98 ± 1.49	0.57 ± 1.17	0.91 ± 1.04	1.06 ± 1.52	0.84 ± 1.24
	*Tannerellaceae*	0.99 ± 3.03	3.47 ± 9.52	0.25 ± 0.76	2.02 ± 4.77	1.97 ± 6.19
	*Veillonellaceae*	0.42 ± 0.42	4.50 ± 6.67	3.11 ± 4.39	1.78 ± 1.25	3.13 ± 4.65
28	*Akkermansiaceae*	1.25 ± 4.15	0.00 ± 0.00	3.06 ± 9.19	0.00 ± 0.00	0.95 ± 5.12
	*Bacteroidaceae*	33.09 ± 25.29	17.19 ± 24.27	14.03 ± 22.19	20.40 ± 23.20	17.31 ± 22.58
	*Bifidobacteriaceae*	21.51 ± 27.70	51.59 ± 22.85	57.09 ± 22.62	46.66 ± 18.45	51.60 ± 21.02
	*Burkholderiaceae*	1.26 ± 3.96	0.11 ± 0.36	0.54 ± 1.63	2.23 ± 3.70	0.98 ± 2.47
	*Clostridiaceae*	5.10 ± 9.64	1.55 ± 2.56	1.09 ± 2.06	4.23 ± 6.76	2.33 ± 4.48
	*Enterobacteriaceae*	22.34 ± 17.71	17.05 ± 14.31	14.12 ± 8.32	12.23 ± 5.88	14.48 ± 10.04
	*Enterococcaceae*	0.12 ± 0.18	1.28 ± 1.67	1.35 ± 1.46	1.61 ± 2.59	1.41 ± 1.92
	*Erysipelotrichaceae*	2.68 ± 6.59	0.08 ± 0.16	0.16 ± 0.34	0.35 ± 0.47	0.20 ± 0.35
	*Lachnospiraceae*	6.05 ± 10.30	0.85 ± 1.49	1.35 ± 1.81	1.69 ± 2.01	1.30 ± 1.75
	*Pasteurellaceae*	0.30 ± 0.65	0.35 ± 0.80	0.22 ± 0.52	0.05 ± 0.08	0.21 ± 0.55
	*Streptococcaceae*	0.17 ± 0.33	0.79 ± 1.02	0.65 ± 0.68	0.28 ± 0.42	0.57 ± 0.75
	*Tannerellaceae*	2.10 ± 6.01	2.48 ± 6.84	0.52 ± 1.08	2.55 ± 5.97	1.89 ± 5.26
	*Veillonellaceae*	0.90 ± 1.65	4.28 ± 5.62	3.26 ± 4.97	5.11 ± 5.64	4.25 ± 5.29
35	*Akkermansiaceae*	1.83 ± 5.78	0.00 ± 0.00	0.93 ± 2.76	0.00 ± 0.00	0.29 ± 1.54
	*Bacteroidaceae*	38.80 ± 24.66	19.89 ± 25.81	21.91 ± 28.26	24.14 ± 24.01	21.99 ± 25.11
	*Bifidobacteriaceae*	16.80 ± 12.25	50.41 ± 21.02	50.31 ± 24.95	43.28 ± 11.70	47.92 ± 19.38
	*Burkholderiaceae*	0.99 ± 2.92	0.00 ± 0.00	0.53 ± 1.50	2.07 ± 3.45	0.88 ± 2.30
	*Clostridiaceae*	7.41 ± 13.62	0.93 ± 1.77	0.76 ± 0.89	1.73 ± 2.86	1.15 ± 2.01
	*Enterobacteriaceae*	18.13 ± 16.05	14.69 ± 11.86	16.04 ± 12.02	14.16 ± 8.44	14.93 ± 10.49
	*Enterococcaceae*	0.41 ± 0.73	0.84 ± 0.90	1.29 ± 1.54	1.74 ± 1.96	1.29 ± 1.52
	*Erysipelotrichaceae*	3.23 ± 7.70	0.13 ± 0.20	0.06 ± 0.15	0.22 ± 0.31	0.14 ± 0.23
	*Lachnospiraceae*	7.55 ± 13.00	0.93 ± 1.40	1.29 ± 1.91	1.76 ± 2.21	1.33 ± 1.83
	*Pasteurellaceae*	0.17 ± 0.36	0.48 ± 0.60	0.00 ± 0.01	1.38 ± 2.85	0.64 ± 1.75
	*Streptococcaceae*	0.61 ± 1.64	0.81 ± 1.10	0.56 ± 0.65	0.95 ± 1.32	0.78 ± 1.05
	*Tannerellaceae*	0.83 ± 1.84	4.31 ± 11.73	0.66 ± 1.99	1.41 ± 3.99	2.18 ± 7.28
	*Veillonellaceae*	0.71 ± 0.88	4.74 ± 7.09	3.05 ± 4.46	4.16 ± 4.36	4.02 ± 5.33
42	*Akkermansiaceae*	0.00 ± 0.00	0.00 ± 0.00	9.10 ± 18.50	0.00 ± 0.00	2.82 ± 10.78
	*Bacteroidaceae*	35.38 ± 26.80	12.16 ± 18.16	12.37 ± 21.87	21.74 ± 26.22	15.53 ± 22.02
	*Bifidobacteriaceae*	20.67 ± 16.13	61.73 ± 19.95	56.15 ± 23.69	45.55 ± 16.25	54.42 ± 20.53
	*Burkholderiaceae*	1.12 ± 2.97	0.00 ± 0.00	0.55 ± 1.65	1.25 ± 2.26	0.60 ± 1.64
	*Clostridiaceae*	5.74 ± 14.61	0.87 ± 1.06	1.10 ± 1.54	1.52 ± 3.30	1.17 ± 2.15
	*Enterobacteriaceae*	18.46 ± 15.23	12.30 ± 6.93	11.76 ± 4.54	12.60 ± 9.44	12.24 ± 7.08
	*Enterococcaceae*	0.30 ± 0.37	0.73 ± 1.18	1.12 ± 0.93	1.55 ± 1.67	1.13 ± 1.31
	*Erysipelotrichaceae*	1.83 ± 3.84	0.08 ± 0.22	0.03 ± 0.10	0.15 ± 0.23	0.09 ± 0.19
	*Lachnospiraceae*	8.65 ± 16.40	1.34 ± 2.28	0.89 ± 1.89	1.13 ± 1.44	1.13 ± 1.84
	*Pasteurellaceae*	0.12 ± 0.26	1.00 ± 2.70	0.00 ± 0.00	3.99 ± 6.47	1.72 ± 4.33
	*Streptococcaceae*	0.18 ± 0.26	0.61 ± 0.65	0.46 ± 0.43	0.25 ± 0.34	0.44 ± 0.50
	*Tannerellaceae*	1.65 ± 4.67	1.93 ± 5.15	0.76 ± 2.28	3.08 ± 8.26	1.96 ± 5.73
	*Veillonellaceae*	0.52 ± 0.59	3.46 ± 5.75	3.24 ± 3.93	4.45 ± 4.32	3.73 ± 4.62
63	*Akkermansiaceae*	0.65 ± 2.05	0.05 ± 0.17	5.12 ± 12.62	0.00 ± 0.00	1.61 ± 7.16
	*Bacteroidaceae*	39.66 ± 26.47	16.29 ± 22.36	19.40 ± 26.16	19.68 ± 23.94	18.42 ± 23.30
	*Bifidobacteriaceae*	22.45 ± 14.56	47.80 ± 14.65	46.56 ± 21.83	51.56 ± 26.41	48.71 ± 20.83
	*Burkholderiaceae*	0.82 ± 2.07	0.02 ± 0.06	0.17 ± 0.51	0.83 ± 1.67	0.34 ± 1.05
	*Clostridiaceae*	0.81 ± 1.48	1.74 ± 2.54	1.32 ± 1.58	0.53 ± 1.11	1.19 ± 1.86
	*Enterobacteriaceae*	15.01 ± 11.55	17.53 ± 11.34	18.18 ± 12.38	13.71 ± 16.63	16.41 ± 13.34
	*Enterococcaceae*	0.32 ± 0.63	1.31 ± 1.28	1.15 ± 1.45	0.72 ± 0.60	1.06 ± 1.15
	*Erysipelotrichaceae*	0.73 ± 0.98	0.08 ± 0.14	0.05 ± 0.11	0.10 ± 0.14	0.08 ± 0.13
	*Lachnospiraceae*	9.38 ± 16.83	1.35 ± 2.10	1.09 ± 1.55	2.18 ± 3.55	1.55 ± 2.53
	*Pasteurellaceae*	0.10 ± 0.13	0.39 ± 0.75	0.13 ± 0.27	2.06 ± 5.13	0.89 ± 3.07
	*Streptococcaceae*	0.18 ± 0.40	0.23 ± 0.45	0.59 ± 0.67	1.36 ± 1.54	0.73 ± 1.09
	*Tannerellaceae*	1.63 ± 4.32	3.86 ± 9.95	0.33 ± 0.77	3.27 ± 8.52	2.56 ± 7.60
	*Veillonellaceae*	3.82 ± 9.18	6.75 ± 7.68	3.64 ± 3.64	1.79 ± 1.55	4.07 5.29

^*a*^ EVC, the mean across all EVC001 doses.

## DISCUSSION

The infant gut microbiome undergoes a profound change in diversity and stability in the first few months of life, becoming more complex over time, and its maturation is influenced by several host factors including infant diet (13). Although we have previously demonstrated that *B. infantis* supplemented to breastfed infants within the first week of life restores the gut microbiome 1 month post-supplementation and persists up to 1 year postnatal (19, 20), no studies have shown this effect in older infants whose gut microbiomes are transitioning toward a mature and more stable gut microbiome (13, 37, 38). Additionally, we explored whether lower doses of the probiotic would be as effective as higher doses, as previously reported. Therefore, this study was designed to determine the effect of a *B. infantis* probiotic supplementation in exclusively breastfed infants aged 2–4 months on fecal *B. infantis* levels and to determine the minimally effective dose required to significantly increase fecal *B. infantis* abundance*.*

The current study showed that *B. infantis* EVC001 supplementation for 28 days significantly increased fecal *B. infantis* abundance in 2-4-month-old infants compared with baseline and led to persistent colonization 1 month post-supplementation. To date, only a paucity of studies in preterm and term infants show colonization of the gut with probiotic supplements containing different species of *Bifidobacterium* when supplemented in the early postnatal period (39–42). To our knowledge, this is the first study investigating the effect of probiotic supplementation in older infants who have a more mature and stable gut microbiome compared with preterm or term newborns (13, 37, 38, 43).

All supplement doses (10^9^ to 10^10^ CFU/day) studied increased fecal *B. infantis* levels significantly when compared with placebo, and there were no differences in fecal *B. infantis* levels between the low-, medium-, and high-dose groups. These results are consistent with a recent double-blind, randomized, placebo-controlled trial that found significant increased relative abundance of fecal *Bifidobacterium* in breastfed term infants supplemented with a different strain of *B. infantis* at a dose of 1 billion CFU/day and was positively correlated with breastfeeding frequency (42). By contrast, fecal recovery of probiotics appears to be dose-dependent in adults at doses ranging from 10^8^ to 10^11^ CFU/day (44–48) and is likely due to the comparatively high level of colonization resistance observed in adulthood (49). Taken together, the unstable gut microbiota in infancy represents a unique opportunity to recolonize the infant gut through the co-evolutionary relationship between keystone infant gut symbionts and human milk.

The most profound difference observed in overall microbiome composition was the increased relative abundance of *Bifidobacteriaceae* from baseline in the EVC group, which was nearly 2-fold higher across each time point compared with the placebo-control group. *Enterococcaceae* was also enriched during and after feeding with *B. infantis*. Notably, members of the *Bifidobacterium* and *Enterococcus* genera were the primary drivers of increased *Bifidobacteriaceae* and *Enterococcaceae,* respectively*.* Conversely, *B. infantis* supplementation also decreased populations of *Enterobacteriaceae* and *Erysipelotrichaceae* during and after supplementation, whereas *Lachnospiraceae* and *Pasteurellaceae* were significantly diminished during the supplementation period. It should be noted that the observed changes in the relative abundances may in part reflect the compositional nature of 16 s rRNA sequencing data, whereby an increase in the relative abundance of one taxon (e.g., *Bifidobacteriaceae* following *B. infantis* supplementation) can mathematically lead to a decrease in others, even without a true decline in their absolute abundance. Future studies using absolute quantification methods (e.g., qPCR) are needed to confirm whether *Enterobacteriaceae* abundance truly decreases following *B. infantis* supplementation.

Consistent with the difference in relative abundance of fecal microbiome measured by 16S rRNA sequencing, beta diversity measured by weighted UniFrac distances was significantly different between the EVC001 supplemented and control groups. Despite these compositional differences in the microbial community, the Shannon diversity index was not significantly different between placebo and EVC001-fed infants, suggesting both microbial richness and evenness were similar across groups. Both the compositional assessments and diversity-related measures are consistent with findings from Frese et al. (2017), with supplementation of EVC001 in breastfed newborns (19).

Probiotic supplementation is reported to be well-tolerated in preterm and term infants (36, 50). In this study, we found no differences in the number of stools; stool size or consistency; number of sleep or crying hours; incidences of cold, runny nose, or cough; fever at or above 103°F; blood in stool; prolonged abdominal bloating or straining; vomiting; or diaper rash between treatment groups (all doses) compared with placebo or baseline, indicating that all *B. infantis* EVC001 doses were well tolerated. The number of infant stools during the supplementation and post-supplementation periods was significantly lower in infants receiving any dose of *B. infantis* EVC001, when compared with baseline. These data are consistent with those of Smilowitz et al., which found a 40% decrease in the number of daily stools in term, breastfed infants supplemented with a high dose of *B. infantis* EVC001 (36). Further analysis of this cohort showed that fecal HMO concentrations were 10-fold lower in breastfed infants supplemented with *B. infantis* EVC001 compared with the control group (19). As HMOs are osmolytes, their high concentrations in the colon observed in infants with low bifidobacterial-HMO utilization capacity result in osmotic perturbation prevalent in malabsorption (51) and explain in part the high frequency in stool number in infants who are not colonized with *B. infantis*.

Early in life, the infant gut microbiome is highly variable among individuals and across time (37, 52). In this study, fecal *B. infantis* levels in the placebo group were highly variable during the progression of the study. Fecal *B. infantis* was undetectable in two placebo-control infants at baseline, but levels had increased to 8 logs during the post-supplementation period. Further investigation led to the discovery that one of these placebo-control infants spent a substantial amount of time with a mother-infant dyad randomized to the medium-dose group. Additionally, in the medium-dose group, two infants had high levels of fecal *B. infantis* at baseline that could not be readily explained. These observations may be explained by the horizontal transfer of *B. infantis* observed among breastfed infants recently shown by Taft and colleagues (10). The horizontal transfer of the *B. infantis* strain EVC001 was found in two infants randomized to the control group of a previously published study (19). Both control infants’ gut microbiomes were devoid of *B. infantis* throughout the duration of the 2-month supplementation trial, yet following a nanny-share and play groups with two different infants randomized to the EVC001 supplemented group, levels of *B. infantis* EVC001 in the two control infants reached 9 logs during the 1 year follow-up study (20). Additionally, sibling presence in the home is known to influence the composition of the infant gut microbiome and several studies have demonstrated that infants with older siblings are more likely to have a *Bifidobacterium* dominant fecal profile compared with infants without siblings in the home (53, 54).

One infant in the EVC group consumed cephalosporin antibiotics between study days 31–38 to treat eczema she was diagnosed with prior to enrolling in the study. It is well known that bifidobacteria are susceptible to cephalosporins (55), and in this infant, fecal *B. infantis* levels decreased on day 35 and rebounded on day 45, 1 week after antibiotic treatment was complete. It is likely that the continued consumption of the EVC001 *B. infantis* supplement with a human milk diet enabled *B. infantis* to persist in the gut and reestablish once treatment was completed. Intake of probiotics and antibiotics simultaneously is likely beneficial as probiotics have been shown to be effective at preventing antibiotic-associated diarrhea in both children and adults (56–58). In very-low birth-weight infants who received post-natal antibiotics, probiotics have been shown to be beneficial; infants who consumed *Lactobacillus acidophilus* or *B. infantis* probiotics following antibiotic treatment showed increased growth rates compared with infants who did not receive probiotics (59).

This study was designed to explore the effects of a daily probiotic supplement on the microbial composition in term infants 2–4 months old and to determine the minimally effective dose to increase fecal *B. infantis* levels above baseline. This was a randomized, double-blind, placebo-controlled trial that followed infants for 1 month post-supplementation, which allowed us to determine persistence of *B. infantis* after supplementation ceased. One limitation is that this study included a small number of participants per group (*n* = 10) and was not powered to determine the effect of the probiotic supplement on other outcomes such as stooling patterns and health conditions. Although our study was powered to detect differences in fecal *B. infantis* in response to EVC001 supplementation, small sample sizes are at risk for reduced generalizability and overfitting predictive models. Additionally, the study followed participants for only 1 month post-supplementation; thus, we do not know if the observed changes on microbial composition will persist long-term. Finally, this study only consisted of infants who were exclusively breastfed, and future studies should focus on whether *B. infantis* can colonize the gut of infants mixed-fed with human milk and formula, which would represent a larger infant population in the United States.

In conclusion, the findings of this study demonstrated that supplementation with *B. infantis* EVC001 for 28 days in 2- to 4-month-old, exclusively breastfed infants across three dosing regimens was well-tolerated and resulted in increased fecal *B. infantis* levels that persisted to at least 1 month post-supplementation. All doses studied significantly increased fecal *B. infantis* levels above baseline in older infants who have a more stable microbiome than newborns. All infants were exclusively breastfed throughout the entire study period, supporting the hypothesis that the persistent colonization we observed is largely due to the unique ability of *B. infantis* to utilize HMOs.

## Data Availability

Sequencing data generated in this study are publicly deposited in the NCBI SRA under BioProjectID accession number PRJNA1188647.

## SUPPLEMENTAL MATERIAL

Supplemental Figures msphere.00518-25-s0001.pdf Figures S1 to S6.

Supplemental Tables msphere.00518-25-s0002.xlsx Tables S1 to S4.

ASM does not own the copyrights to Supplemental Material that may be linked to, or accessed through, an article. The authors have granted ASM a non-exclusive, world-wide license to publish the Supplemental Material files. Please contact the corresponding author directly for reuse.
