# Supplementary material for: Randomized, placebo-controlled trial reveals the impact of dose and timing of Bifidobacterium infantis probiotic supplementation on breastfed infants’ gut microbiome
Source: mSphere. 2025 Dec 22;11(1):e00518-25. doi: 10.1128/msphere.00518-25 (PMC12838401; doi:10.1128/msphere.00518-25)
Supplement: Supplemental Figures — Figures S1 to S6. [file msphere.00518-25-s0001.pdf]

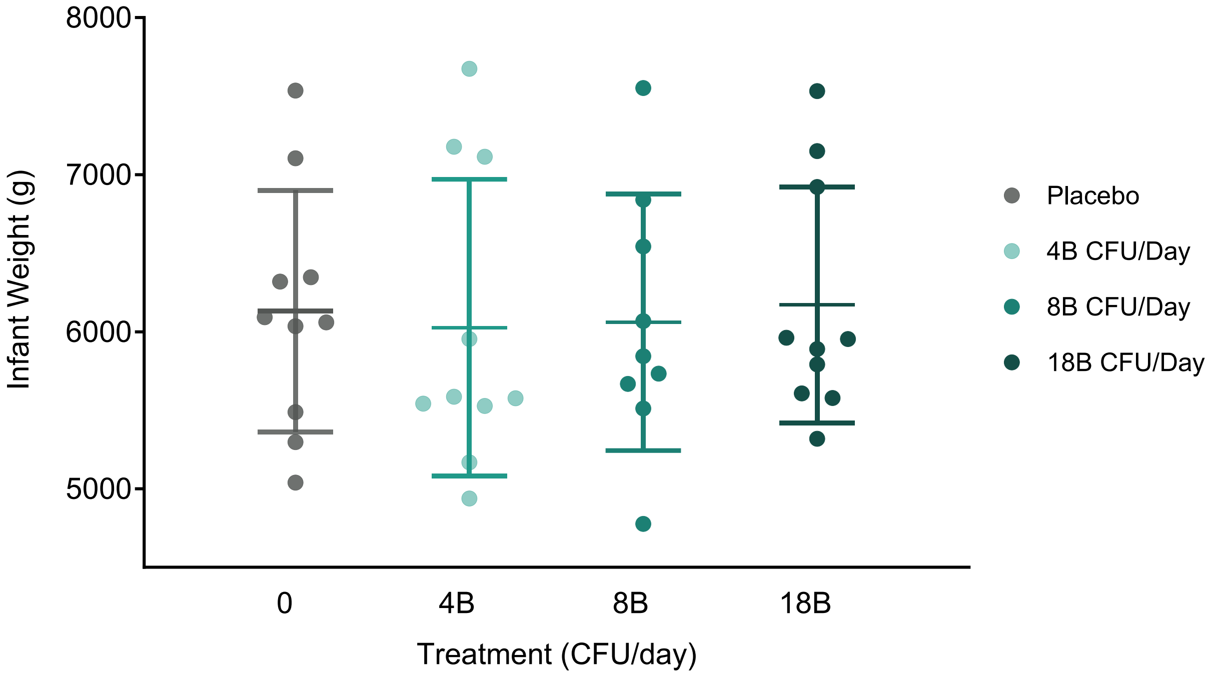

Supplemental Figure 1. Infant weight (g) at Baseline by treatment group.

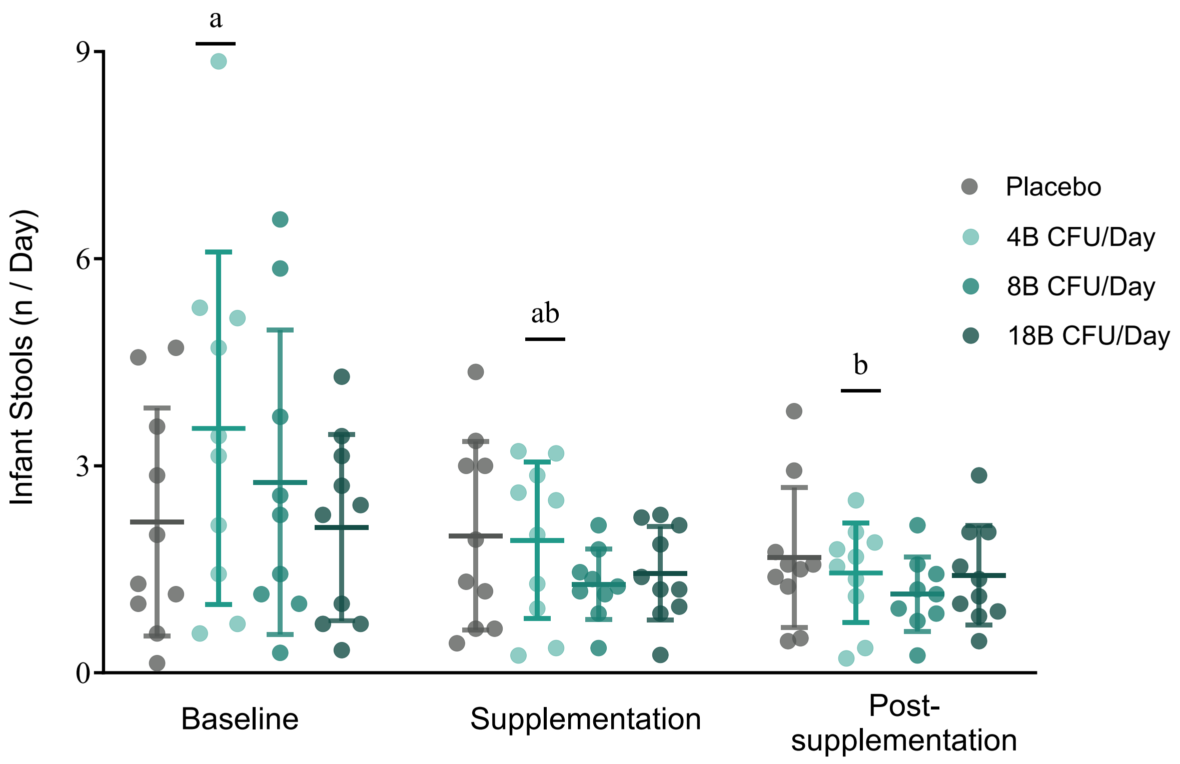

Supplemental Figure 2. Number of infant stools across time and inclusive of all dose groups, Bonferroni adjusted $P < 0.01$ for differences between Baseline and Post-supplementation within the low dose (4B CFU/day) group (Friedman test).

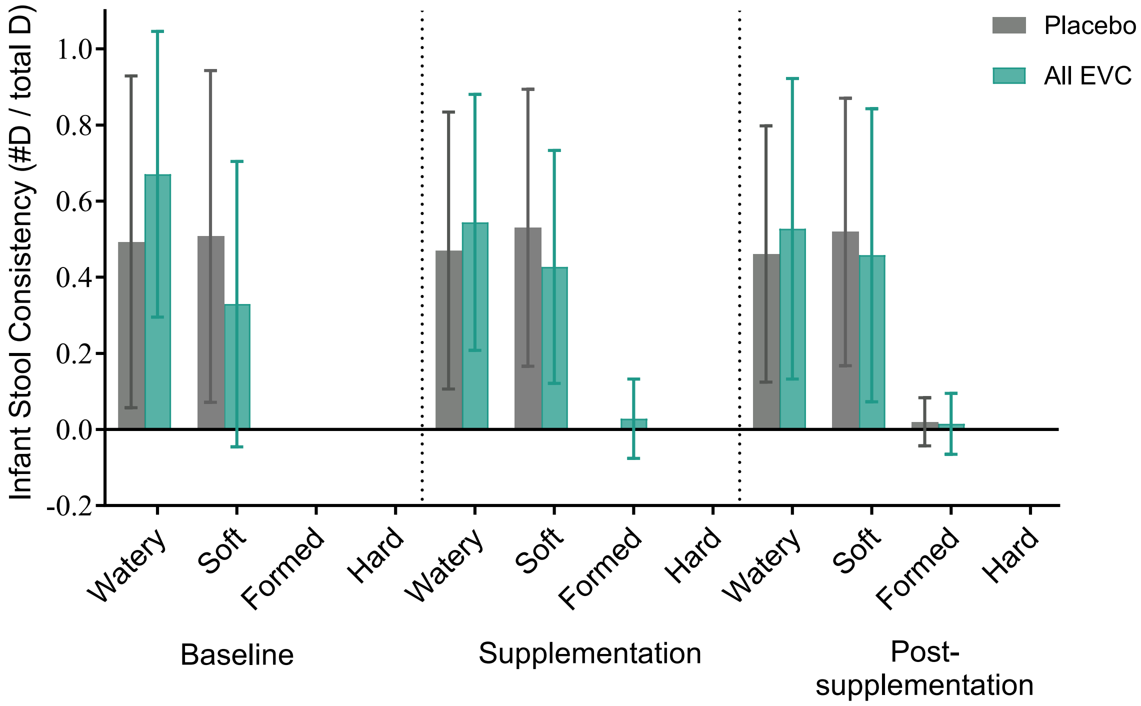

Supplemental Figure 3. Mean proportion of infant stool consistencies during the Baseline (Days 1-7), Supplementation (Days 8-35), and Post-Supplementation (Days 36-65) periods.

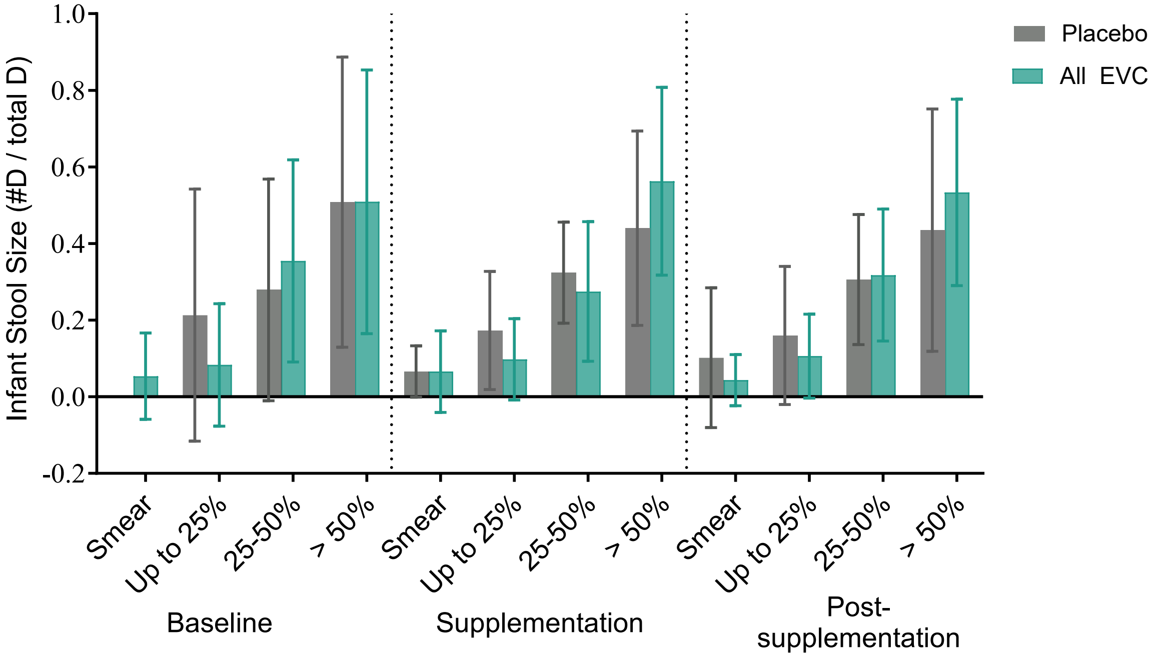

Supplemental Figure 4. Mean proportion of infant stool sizes during the Baseline (Days 1-7), Supplementation (Days 8-35), and Post-Supplementation (Days 36-65) periods.

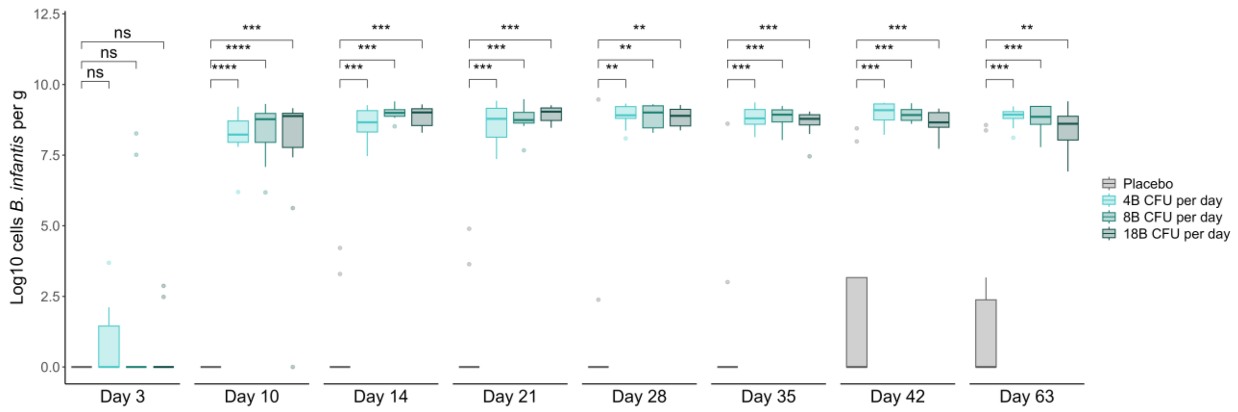

Supplemental Figure 5. Infant fecal *B. infantis* across time and between treatment groups. ns = not significant, ** $P < 0.01$, **** $P < 0.0005$ for differences in the ranks between each treatment group compared with placebo at each time point.

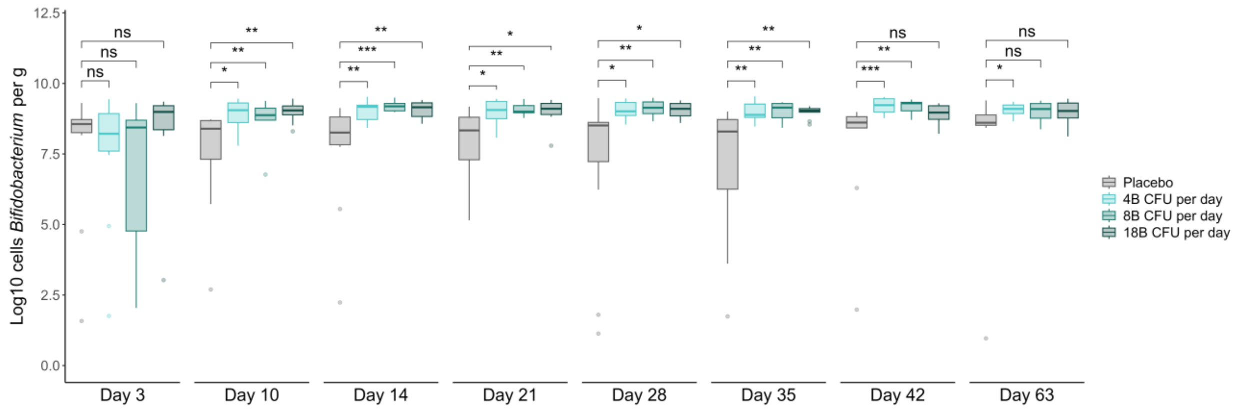

Supplemental Figure 6. Total fecal *Bifidobacterium* across time and among treatment groups. * $P < 0.05$, ** $P < 0.01$, **** $P < 0.0005$, ns = not significant for differences in the ranks between each treatment group compared with placebo at each time point**.**
